# A comparison of the diagnostic ability of vessel density and structural measurements of optical coherence tomography in primary open angle glaucoma

**DOI:** 10.1371/journal.pone.0173930

**Published:** 2017-03-13

**Authors:** Harsha L. Rao, Zia S. Pradhan, Robert N. Weinreb, Mohammed Riyazuddin, Srilakshmi Dasari, Jayasree P. Venugopal, Narendra K. Puttaiah, Dhanaraj A. S. Rao, Sathi Devi, Kaweh Mansouri, Carroll A. B. Webers

**Affiliations:** 1 Narayana Nethralaya, Rajajinagar, Bangalore, India; 2 Narayana Nethralaya, Hulimavu, Bangalore, India; 3 Shiley Eye Institute, Hamilton Glaucoma Center and Department of Ophthalmology, University of California San Diego, San Diego, La Jolla, California, United States of America; 4 Glaucoma Center, Montchoisi Clinic, Swiss Vision Network, Lausanne, Switzerland; 5 Department of Ophthalmology, University of Colorado, Denver, Colorado, United States of America; 6 University Eye Clinic Maastricht, University Medical Center, Maastricht, the Netherlands; Bascom Palmer Eye Institute, UNITED STATES

## Abstract

**Purpose:**

To compare the diagnostic abilities of vessel density measurements of the optic nerve head (ONH), peripapillary and macular regions on optical coherence tomography (OCT) angiography in eyes with primary open angle glaucoma (POAG) with that of the ONH rim area, peripapillary retinal nerve fiber layer (RNFL) thickness and the macular ganglion cell complex (GCC) thickness measurements.

**Methods:**

In a cross sectional study, 78 eyes of 50 control subjects and 117 eyes of 67 POAG patients underwent vessel density and structural measurements with spectral domain OCT. POAG was diagnosed based on the masked evaluation of optic disc stereo photographs. Area under receiver operating characteristic curves (AUC) and sensitivities at fixed specificities of vessel densities in ONH, peripapillary and macular regions were compared with rim area, RNFL and GCC thickness.

**Results:**

The AUC (sensitivity at 95% specificity) of average vessel densities within the ONH, peripapillary and macular region were 0.77 (31%), 0.85 (56%) and 0.70 (18%) respectively. The same of ONH rim area, average RNFL and GCC thickness were 0.94 (83%), 0.95 (72%) and 0.93 (62%) respectively. AUCs of vessel densities were significantly lower (p<0.05) than that of the corresponding structural measurements. Pre-treatment IOP (coefficient: 0.08) affected (p<0.05) the AUC of ONH vessel density but not of any other vessel density or structural measurements.

**Conclusions:**

Diagnostic abilities of ONH, peripapillary and the macular vessel densities in POAG were significantly lower than ONH rim area, peripapillary RNFL and macular GCC measurements respectively. At fixed levels of glaucoma severity, the diagnostic ability of the ONH vessel density was significantly greater in eyes with higher pre-treatment IOP.

## Introduction

Optical coherence tomography (OCT) angiography is a new technique of non-invasively imaging the blood vessels of the optic nerve head (ONH) and retina in-vivo. Of the multiple algorithms developed to achieve blood vessel delineation using the OCT platform, split spectrum amplitude-decorrelation angiography (SSADA) was the first one that was commercially available.[1] Early studies using the SSADA algorithm have shown that the vessel density measurements provided by OCT angiography (OCTA) were repeatable and reproducible.[2–6].

Primary open angle glaucoma (POAG) is a chronic progressive optic neuropathy resulting from the apoptosis of the retinal ganglion cells (RGC).[7] Evaluating the neuroretinal rim area, retinal nerve fiber layer (RNFL) and ganglion cell complex (GCC) thickness on OCT is used as a surrogate measure in clinical practice to estimate the amount of RGC loss in POAG. Although increased intraocular pressure (IOP) is the predominant risk factor for RGC death,[8] reduced ONH perfusion has also been proposed to play a role in the pathogenesis of glaucoma.[9, 10] Studies using the SSADA algorithm of OCTA have demonstrated reduced ONH and peripapillary vessel densities in patients with glaucoma.[2–5, 11, 12] In addition to reduced ONH and peripapillary vessel densities, we recently demonstrated reduced vessel densities in the macula of patients with POAG.[13] Previous studies have compared the diagnostic ability of peripapillary vessel density measurements of OCTA with the RNFL thickness measurements of OCT.[5, 12] However, to the best of our knowledge, there are no studies comparing the diagnostic ability of inside disc vessel densities with ONH rim area or the macular vessel density with macular GCC thickness. The purpose of the current study was to compare the diagnostic abilities of the vessel density measurements of the ONH, peripapillary and macular regions on OCTA in eyes with POAG with that of the ONH rim area, peripapillary RNFL thickness and the macular GCC thickness measurements on OCT. The secondary objective was to evaluate the effect of pre-treatment IOP on the diagnostic abilities of the vessel densities and structural measurements of OCT.

## Methods

This was a prospective, cross-sectional study conducted at Narayana Nethralaya, a tertiary eye care center in Bengaluru, South India between September 2015 and July 2016. The methodology adhered to the tenets of the Declaration of Helsinki for research involving human subjects. Written informed consent was obtained from all participants and the study was approved by the Ethics Committee of Narayana Nethralaya (approval number: C/2015/08/04).

Participants of the study included control subjects and POAG patients. Control subjects were either hospital staff or subjects who consulted for a routine eye examination or a refractive error. Control subjects had no family history of glaucoma, IOP≤21 mm Hg, open angles on gonioscopy, normal anterior and posterior segment on clinical examination by an ophthalmologist and non-glaucomatous optic discs, as assessed by glaucoma experts on masked examination of stereoscopic optic disc photographs. POAG patients had open angles on gonioscopy and glaucomatous changes on optic nerve head examination (neuroretinal rim narrowing, notching and retinal nerve fiber layer defects) as documented by glaucoma experts on dilated examination and confirmed by experts on stereoscopic optic disc photographs. Neither pre-treatment IOP, nor visual field changes were used to define POAG. Inclusion criteria for all participants were age ≥18 years, corrected distance visual acuity of 20/40 or better and refractive error within ±5 D sphere and ±3 D cylinder. Exclusion criteria were presence of any media opacities that prevented good quality OCT scans, or any retinal or neurological disease other than glaucoma, which could confound the evaluation. Eyes with a history of trauma or inflammation were also excluded. All participants underwent a comprehensive ocular examination, which included a detailed medical history, corrected distance visual acuity measurement, slit-lamp biomicroscopy, Goldmann applanation tonometry, gonioscopy, dilated fundus examination, visual field (VF) examination and OCT imaging with RTVue-XR SD-OCT (Optovue Inc., Fremont, CA). In addition to IOP measured on the day of scanning, the pre-treatment IOP (i.e. the IOP noted on the day on initiating anti-glaucoma treatment) was documented for all POAG eyes.

Stereoscopic optic disc photographs were obtained by trained technicians using a digital fundus camera (Kowa nonmyd WX, Kowa Company, Ltd., Japan). Each optic disc photograph was evaluated independently by two glaucoma experts (HLR and NKP) in a masked manner to determine the presence of glaucomatous changes (focal or diffuse neuroretinal rim thinning, localized notching or RNFL defects). The experts were masked to all the clinical data, visual field data and the fellow eye data. Discrepancy in the classification between the two experts was adjudicated by a third glaucoma expert (ZSP).

VF examination was performed using a Humphrey Field analyzer II, model 720i (Zeiss Humphrey Systems, Dublin, CA), with the Swedish interactive threshold algorithm (SITA) standard 24–2 program. VFs were considered reliable if the fixation losses were less than 20%, and the false positive and false negative response rates were less than 15%. VF findings were not used for defining glaucoma or controls but were considered for the grading of glaucoma severity.

OCTA imaging of the optic disc region and macula was performed using RTVue-XR SD-OCT (AngioVue, v2015.100.0.33). The procedure of OCTA imaging with RTVue-XR has been detailed previously.[13] In brief, it uses an 840 nm diode laser source, with an A-scan rate of 70 kHz per second. Imaging is performed using a set of 2 scans; one vertical priority and one horizontal priority raster volumetric scan. The optic disc scan covers an area of 4.5 × 4.5 mm and the macular scan was performed using volumetric scans covering 3 x 3 mm. An orthogonal registration algorithm is used to produce merged 3-dimensional OCT angiograms.[14] The SSADA algorithm compares the consecutive B-scans at the same location to detect flow using motion contrast, thereby delineating blood vessels.[1] Vessel density is defined as the percentage area occupied by the large vessels and microvasculature in a particular region. Vessel densities are calculated over the entire scan area, i.e. whole enface disc and whole enface macula, as well as defined areas within each scan as described below. In addition, the software calculates vessel densities in various layers of the retina and the ONH.

In the optic disc scan, the software automatically fits an ellipse to the optic disc margin and calculates the average vessel density within the ONH (referred to as the inside disc vessel density). The peripapillary region is defined as a 0.75 mm-wide elliptical annulus extending from the optic disc boundary and the average vessel density with this region is calculated. Both the ONH and the peripapillary region are divided into 6 sectors based on the Garway-Heath map and the vessel densities in each sector is calculated (nasal, inferonasal, inferotemporal, superotemporal, superonasal and temporal sectors).[15] In order to compare the inside disc and peripapillary vessel densities with the rim area and the RNFL measurements of OCT, the superonasal and superotemporal sectors were combined together as the superior sector, and the inferonasal and inferotemporal sectors as the inferior sector. For each scanned region, the software calculates the vessel densities in various layers of the retina and ONH. For the purpose of this study, the antero-posterior segment used for each region is as follows. The ONH vessel densities were calculated from the “nerve head segment” of the ONH angiogram. This segment extends from 2000 microns above the internal limiting membrane (ILM) to 150 microns below the ILM. The peripapillary vessel density was analyzed from the “Radial Peripapillary Capillary (RPC) segment” which extends from the ILM to the posterior boundary of the nerve fiber layer. Macular vessel densities were analyzed over a 1.5 mm-wide parafoveal, circular annulus centered on the macula. The parafoveal region was also divided into 4 sectors of 90° each (nasal, inferior, superior and temporal sectors). Macular vessel densities analyzed in this study were of the superficial vascular plexus present in the inner layers of the retina (extending from the internal limiting membrane to the inner plexiform layer).

**Fig 1:**
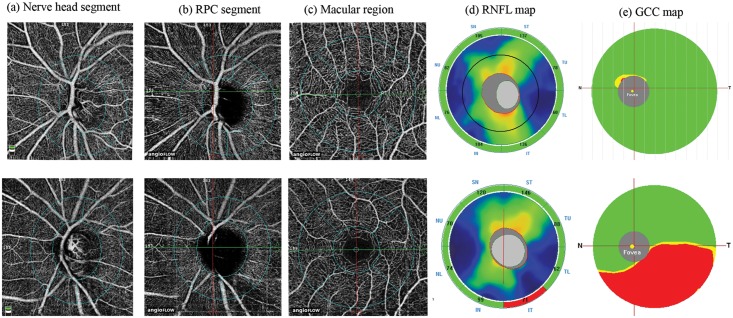
Case examples. Nerve head segment (a), radial peripapillary capillary, RPC segment (b) and macular (c) optical coherence tomography angiography scans of a normal eye (top panel) and an eye with glaucoma (bottom panel). The figure also shows the retinal nerve fiber layer, RNFL (d) and ganglion cell complex, GCC (e) maps of the two eyes. Vessel loss in the eye with glaucoma can be noted in the inferotemporal peripapillary region correlating with the RNFL loss seen on the RNFL map.

All subjects also underwent the traditional ONH, peripapillary RNFL and macular GCC thickness measurements on RTVue-XR SD-OCT using the ONH and the GCC scans. These scan protocols have been explained in detail previously.[16, 17] All the examinations for a particular subject were performed on the same day. Image quality was assessed for all OCTA and OCT scans. Poor quality images, which were defined as those with a signal strength index (SSI) less than 35 or images with motion artifacts and segmentation errors were excluded from the analysis. Fig 1 shows the OCTA and the OCT maps of a normal eye and an eye with POAG.

## Statistical analysis

Descriptive statistics included mean and standard deviation for normally distributed variables and median and inter-quartile range (IQR) for non-normally distributed variables. Shapiro-Wilk test was used to test for the normality distribution of continuous variables. Normally distributed continuous variables between the control and the glaucoma groups were compared using t test. Non-normally distributed continuous variables were compared using Wilcoxon rank sum test. Percentages were compared using Chi square test. Receiver operating characteristic (ROC) curves were used to describe the ability of vessel density and structural measurements of OCT to discriminate glaucomatous eyes from control eyes. Sensitivities at fixed specificities of 80% and 95% were determined for all the parameters. To obtain confidence intervals for area under the ROC curves (AUC) and sensitivities, a bootstrap re-sampling procedure was used (n = 1000 re-samples). As measurements from both eyes of the same subject are likely to be correlated, the standard statistical methods for parameter estimation can lead to underestimation of standard errors and to confidence intervals that are too narrow.[18] Therefore, the cluster of data for the study subject was considered as the units of resampling and bias corrected standard errors were calculated during all estimations. This procedure has been used to adjust for the presence of multiple correlated measurements from the same unit.[19, 20] To compare the AUCs, a Wald statistic, dividing the observed AUC difference by its standard error, was compared with the standard normal distribution and a p value was reported. ROC regression modeling technique was used to evaluate the effect of glaucoma severity and the pre-treatment IOP on the AUCs and sensitivities of OCT measurements in diagnosing glaucoma.[21, 22].

Statistical analyses were performed using commercial software (Stata ver. 13.1; StataCorp, College Station, TX). A two-tailed p value of ≤0.05 was considered statistically significant.

## Results

Two hundred and twenty-two eyes of 123 subjects (78 eyes of 50 normal and 144 eyes of 73 POAG subjects) underwent vessel density and structural imaging with OCT. Among these, 25 eyes of 18 POAG patients in which the optic disc classification on stereo photographs was not glaucomatous optic neuropathy, were excluded. Of the remaining eyes, 8 eyes with unreliable VF, 19 eyes with poor OCTA scans of ONH, 25 eyes with poor OCTA scans of macula, 17 eyes with poor structural scans of ONH and 2 eyes with poor GCC scans were excluded. Final analysis included vessel density and structural scans from 195 eyes of 117 subjects (78 eyes of 50 normal and 117 eyes of 67 POAG subjects). Of the 117 eyes with POAG, 22 eyes had a “within normal limit” or a “borderline” glaucoma hemifield test result, and / or the probability value of pattern standard deviation >5% on VF (preperimetric glaucoma). Table 1 shows the clinical, VF, vessel density and structural measurements of the included subjects. SSI of the OCTA and structural scan of ONH were significantly greater in the control subjects compared to the POAG patients. AUCs and sensitivities at fixed specificities of optic disc and peripapillary vessel density and structural parameters were therefore calculated after adjusting for the difference in signal strength between the control and POAG groups using covariate-adjustment as proposed by Pepe.[23] All the vessel density and structural measurements were significantly lesser in the glaucoma compared to the control group.

**Table 1:** Clinical features, visual field parameters, vessel density and structural measurements of the participants. All values represent median and interquartile range unless specified.

	Control group (78 eyes, 50 subjects)	POAG group (117 eyes, 67 patients)	P
Age (years)*	60.7 ± 8.3	62.8 ± 12.1	0.30
**Gender (male:female)**	27:23	47:20	0.07
**Sphere (D)**	0.5 (0, 1)	0.0 (-0.75, 0.75)	0.12
**Cylinder (D)**	-0.5 (-1, -0.5)	-0.75 (-1, -0.5)	0.72
**Pseudophakia (n, %)**	13 (16.7%)	29 (24.8%)	0.39
**Optic disc area (mm**^**2**^**)**	2.28 (1.93, 2.53)	2.27 (2.01, 2.59)	0.45
**Pre-treatment IOP (mm Hg)**		20 (18, 24.5)	
**IOP at the scanning visit (mm Hg)**	15.5 (14, 18)	16 (14, 19)	0.001
**Hypertension (yes:no)**	19:31	29:38	0.57
**Diabetes mellitus (yes:no)**	17:33	20:47	0.63
**Mean deviation (dB)**	-0.9 (-3.5, -0.3)	-6.3 (-12.5, -3.5)	<0.001
**Pattern standard deviation (dB)**	1.9 (1.5, 2.5)	4.8 (2.6, 9.7)	<0.001
**Visual field index (%)**	99 (97, 99)	88 (69, 95)	<0.001
**OCTA parameters**			
SSI (Optic disc scan)*	53.6 ± 8.9	49.6 ± 7.8	0.002
**Whole enface vessel density (disc scan, %)**	53.9 (51.3, 55.5)	45.2 (41.5, 48.7)	<0.001
**Inside disc vessel density (%)**	48.1 (44.0, 50.0)	40.1 (34.8, 45.63)	<0.001
**Nasal vessel density (%)**	48.9 (44.1, 52.7)	41.2 (33.6, 47.0)	<0.001
**Inferior vessel density (%)**	48.7 (44.8, 53.3)	43.8 (37.5, 49.5)	<0.001
**Superior vessel density (%)**	49.1 (43.4, 52.2)	40.8 (33.2, 46.2)	<0.001
**Temporal vessel density (%)**	44.3 (40.4, 50.7)	34.9 (28.8, 43.6)	<0.001
**Average Peripapillary vessel density (%)**	61.9 (59.9, 64.2)	54.4 (49.0, 58.6)	<0.001
**Nasal vessel density (%)**	59.5 (57.0, 61.8)	53.2 (46.9, 57.4)	<0.001
**Inferior vessel density (%)**	64.7 (62.1, 67.2)	53.9 (45.6, 59.5)	<0.001
**Superior vessel density (%)**	63.6 (60.3, 66.9)	55.2 (49.4, 60.7)	<0.001
**Temporal vessel density (%)**	60.3 (58.0, 63.2)	56.5 (51.3, 59.8)	<0.001
SSI (Macula scan)*	61.1 ± 6.7	59.9 ± 7.7	0.33
**Whole enface vessel density (macula scan, %)**	47.1 (45.5, 50.1)	43.8 (41.8, 47.0)	<0.001
**Parafoveal vessel density (%)**	49.5 (47.3, 52.3)	46.9 (43.9, 49.4)	<0.001
**Nasal vessel density (%)**	48.3 (46.4, 51.1)	45.9 (43.1, 48.8)	0.001
**Inferior vessel density (%)**	51.0 (48.0, 53.3)	46.6 (43.7, 51.1)	<0.001
**Superior vessel density (%)**	50.3 (48.1, 53.2)	47.5 (44.3, 51.6)	0.002
**Temporal vessel density (%)**	49.6 (46.8, 52.5)	46.8 (44.1, 49.7)	<0.001
**OCT parameters**			
**SSI (ONH scan)**	55.2 ± 8.1	49.4 ± 8.3	<0.001
**Neuroretinal rim area (mm**^**2**^**)**	1.32 (1.12, 1.51)	0.72 (0.56, 0.88)	<0.001
**Nasal rim area (mm**^**2**^**)**	0.40 (0.34, 0.44)	0.21 (0.15, 0.28)	<0.001
**Inferior rim area (mm**^**2**^**)**	0.41 (0.33, 0.48)	0.16 (0.10, 0.26)	<0.001
**Superior rim area (mm**^**2**^**)**	0.38 (0.32, 0.48)	0.22 (0.17, 0.28)	<0.001
**Temporal rim area (mm**^**2**^**)**	0.12 (0.09, 0.19)	0.09 (0.06, 0.14)	<0.001
**Average Peripapillary RNFL thickness (μm)**	100 (93, 105)	79 (70, 85)	<0.001
**Nasal RNFL thickness (μm)**	80 (73, 87)	66 (58, 73)	<0.001
**Inferior RNFL thickness (μm)**	123 (116, 132)	86 (70, 98)	<0.001
**Superior RNFL thickness (μm)**	124 (115, 133)	97 (83, 107)	<0.001
**Temporal RNFL thickness (μm)**	71 (66, 79)	63 (57, 69)	<0.001
SSI (GCC scan)*	59.1 ± 9.7	58.7 ± 9.5	0.78
**Average GCC thickness (μm)**	95 (91, 102)	79 (72, 85)	<0.001
**Superior GCC thickness (μm)**	95 (90, 102)	82 (74, 89)	<0.001
**Inferior GCC thickness (μm)**	96 (91, 102)	77 (66, 84)	<0.001

POAG: primary open angle glaucoma; D: diopter; dB: decibel; IOP: intraocular pressure; SSI: signal strength index; ONH: optic nerve head; RNFL: retinal nerve fiber layer; GCC: ganglion cell complex;

*mean ± standard deviation.

**Fig 2:**
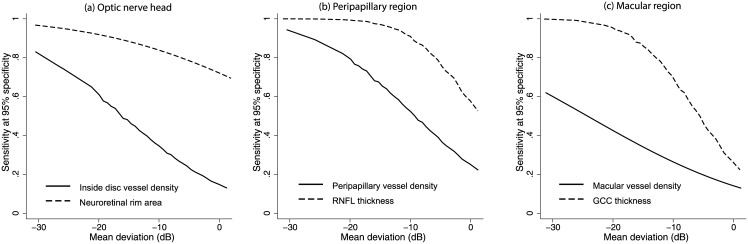
Diagnostic abilities of vessel density and structural measurements. Sensitivity at 95% specificity of (a) optic nerve head vessel density and rim area, (b) peripapillary vessel density and retinal nerve fiber layer (RNFL) thickness and (c) superficial macular vessel density and ganglion cell complex (GCC) thickness according to mean deviation on visual fields.

The AUCs and sensitivities at fixed specificities of the vessel density measurements to differentiate POAG from control eyes are shown in Table 2. Whole enface vessel density of the disc scan showed the best AUC and sensitivity at fixed specificity to diagnose glaucoma. The AUCs and sensitivities at fixed specificities of the structural measurements to differentiate POAG from control eyes are shown in Table 3. Average and the inferior quadrant RNFL thickness showed the best AUC and sensitivity at fixed specificity to diagnose glaucoma. Comparing the diagnostic abilities region-wise, ONH rim area showed a statistically significantly better (p<0.001) AUC than the inside disc vessel density, peripapillary RNFL thickness showed a statistically significantly better (p = 0.002) AUC than the peripapillary vessel density, and macular GCC thickness showed a statistically significantly better (p<0.001) AUC than the macular vessel density. Fig 2 shows the sensitivity at 95% specificity of the vessel density and structural measurements at different severities of glaucomatous VF loss. Sensitivities of structural measurements were better than the vessel densities of the corresponding regions over the whole range of glaucoma severity.

**Table 2:** Diagnostic ability of vessel density parameters of optical coherence tomography angiography in differentiating open angle glaucoma from control eyes (figures in parenthesis represent 95% confidence intervals).

Vessel density	AUC	Sensitivity at 95% specificity	Sensitivity at 80% specificity
**Whole enface (disc scan)**	0.93 (0.88–0.96)	75% (48–89)	88% (75–95)
**Inside disc**	0.77 (0.67–0.86)	31% (08–60)	58% (36–74)
**Nasal**	0.74 (0.65–0.83)	19% (01–41)	57% (28–70)
**Inferior**	0.67 (0.57–0.77)	25% (06–44)	46% (27–61)
**Superior**	0.73 (0.62–0.81)	23% (04–45)	47% (28–69)
**Temporal**	0.70 (0.56–0.81)	13% (03–53)	47% (13–62)
**Average Peripapillary**	0.85 (0.78–0.90)	56% (39–70)	76% (62–87)
**Nasal**	0.78 (0.68–0.85)	40% (29–59)	62% (46–79)
**Inferior**	0.88 (0.81–0.92)	66% (47–83)	78% (66–90)
**Superior**	0.82 (0.73–0.88)	52% (35–68)	66% (51–77)
**Temporal**	0.68 (0.57–0.77)	26% (08–52)	45% (28–57)
**Whole enface (macula scan)**	0.73 (0.64–0.81)	18% (01–40)	57% (37–72)
**Parafoveal**	0.70 (0.61–0.78)	10% (03–36)	49% (34–64)
**Nasal**	0.65 (0.55–0.74)	08% (01–27)	50% (28–64)
**Inferior**	0.69 (0.60–0.77)	19% (04–41)	52% (33–64)
**Superior**	0.65 (0.55–0.74)	15% (03–38)	42% (15–60)
**Temporal**	0.67 (0.58–0.76)	12% (02–26)	42% (18–66)

AUC: area under the receiver operating characteristic curve.

**Table 3:** Diagnostic ability of structural parameters of optical coherence tomography in differentiating open angle glaucoma from control eyes (figures in parenthesis represent 95% confidence intervals).

Vessel density	AUC	Sensitivity at 95% specificity	Sensitivity at 80% specificity
**Neuroretinal rim area**	0.94 (0.88–0.98)	83% (70–95)	89% (78–97)
**Nasal rim area**	0.88 (0.81–0.94)	58% (38–73)	79% (63–89)
**Inferior rim area**	0.92 (0.85–0.96)	73% (57–82)	84% (72–93)
**Superior rim area**	0.89 (0.83–0.94)	65% (50–83)	86% (75–95)
**Temporal rim area**	0.72 (0.61–0.82)	26% (09–38)	52% (34–74)
**Average RNFL thickness**	0.95 (0.91–0.98)	72% (45–88)	94% (85–99)
**Nasal RNFL thickness**	0.83 (0.73–0.90)	44% (03–65)	69% (46–86)
**Inferior RNFL thickness**	0.95 (0.91–0.98)	84% (72–92)	90% (81–96)
**Superior RNFL thickness**	0.90 (0.83–0.94)	56% (38–72)	81% (63–90)
**Temporal RNFL thickness**	0.73 (0.63–0.82)	24% (13–46)	51% (26–69)
**Average GCC thickness**	0.93 (0.88–0.96)	62% (47–83)	87% (75–95)
**Superior GCC thickness**	0.86 (0.79–0.91)	41% (28–63)	77% (61–88)
**Inferior GCC thickness**	0.92 (0.87–0.95)	63% (49–74)	86% (70–93)

AUC: area under the receiver operating characteristic curve; RNFL: retinal nerve fiber layer; GCC: ganglion cell complex.

**Fig 3:**
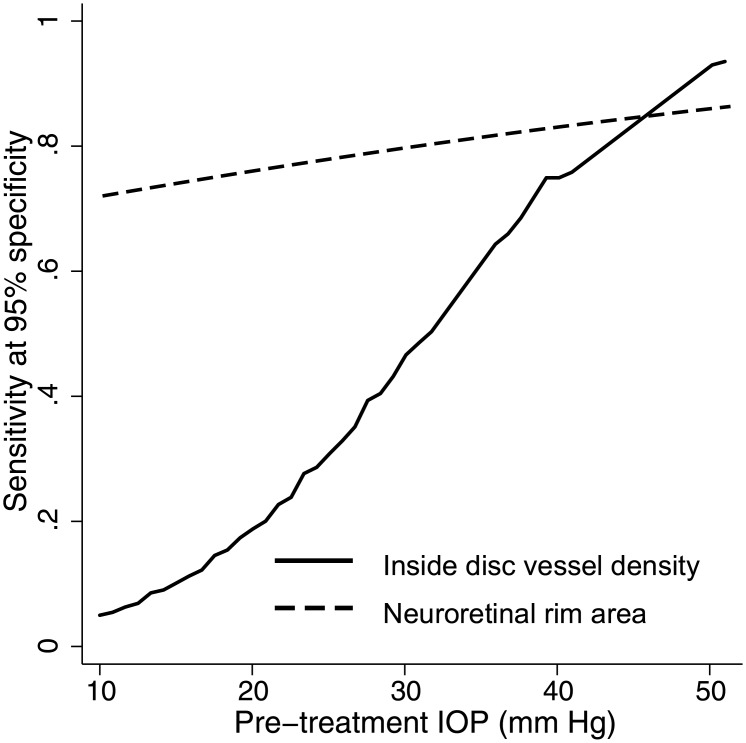
Effect of pre-treatment intraocular pressure on the diagnostic ability of optic nerve head vessel density and rim area. Sensitivity at 95% specificity of optic nerve head vessel density and rim area according to the pre-treatment intraocular pressure at a mean deviation on visual fields of -5 dB.

Table 4 shows the effect of pre-treatment IOP on the diagnostic abilities of vessel density and structural parameters of the three regions, after adjusting for the disease severity as determined by the MD of the VF. Pre-treatment IOP had a significant positive effect on the AUC of inside disc vessel density but not on any other vessel density or structural measurement. AUC and sensitivities at fixed specificities of inside disc vessel density increased significantly in eyes with higher pre-treatment IOPs. Fig 3 shows the effect of pre-treatment IOP on the sensitivities at 95% specificity of the inside disc vessel density and rim area measurements at a MD value of -5 dB.

**Table 4:** Results of the multivariate Receiver Operating Characteristic (ROC) regression models evaluating the effect of mean deviation on visual fields and pre-treatment Intraocular Pressure (IOP) on the area under the ROC curves of the vessel density and structural measurements inside the optic disc, peripapillary and parafoveal region. Figures represent coefficient with 95% confidence interval in parenthesis.

Parameter	Mean deviation	Pre-treatment IOP
**Optic nerve head**		
**Vessel density**	-0.06 (-0.12, -0.01)*	0.08 (0.03, 0.16)*
**Rim area**	-0.04 (-0.12, 0.08)	0.01 (-0.06, 0.23)
**Peripapillary region**		
**Vessel density**	-0.07 (-0.14, -0.01)*	0.02 (-0.02, 0.08)
**RNFL thickness**	-0.14 (-0.34, -0.05)*	-0.06 (-0.22, 0.05)
**Parafoveal region**		
**Vessel density**	-0.05 (-0.10, 0.00)*	-0.01 (-0.04, 0.04)
**GCC thickness**	-0.12 (-0.23, -0.04)*	-0.01 (-0.07, 0.04)

*—statistically significant (p<0.05).

RNFL: retinal nerve fiber layer; GCC: ganglion cell complex.

We ran the entire analysis considering one eye of subjects who contributed both eyes for our earlier analysis and found similar results. When considering the better eye of the glaucoma patients for analysis (median MD: -4.3 dB), the AUC of inside disc (0.74), peripapillary (0.82) and parafoveal (0.70) vessel densities were significantly lower (p<0.01 for all comparisons) than ONH rim area (0.93), peripapillary RNFL (0.93) and average GCC thickness (0.89) respectively. When considering the worse eye of the glaucoma patients for analysis (median MD: -8.5 dB), the AUC of inside disc (0.78), peripapillary (0.88) and parafoveal (0.74) vessel densities were similarly significantly lower (p<0.02 for all comparisons) than ONH rim area (0.93), peripapillary RNFL (0.96) and average GCC thickness (0.95) respectively.

We also ran the entire analysis considering optic disc changes and VF changes as the definition of glaucoma (excluding preperimetric glaucoma eyes) and found similar results. The AUC of inside disc (0.79), peripapillary (0.88) and parafoveal (0.72) vessel densities were still significantly lower (p<0.01 for all comparisons) than ONH rim area (0.96), peripapillary RNFL (0.97) and average GCC thickness (0.96) respectively.

## Discussion

In this study, vessel density measurements of OCTA were compared with structural measurements of the traditional OCT. It was found that the diagnostic abilities of several OCT parameters (ONH rim area, peripapillary RNFL thickness and the macular GCC thickness) in POAG were significantly better than the corresponding vessel densities within each of these regions.

Previous studies have compared the diagnostic ability of peripapillary vessel density measurements of OCTA with the RNFL thickness measurements of OCT.[5, 12] However, to the best of our knowledge, there are no studies comparing the inside disc vessel densities with ONH rim area or the macular vessel densities with macular GCC thickness. Liu et al evaluated the diagnostic ability of peripapillary vessel density and average RNFL thickness in 12 (9 perimetric and 3 pre-perimetric) glaucoma and 12 normal eyes. AUC, sensitivity and specificity of peripapillary vessel density (0.94, 83.3% and 91.7% respectively) was found to be comparable to that of the average RNFL thickness (0.97, 91.7% and 91.7% respectively).[5] Yarmohammadi et al compared the diagnostic ability of peripapillary vessel density with that of the average RNFL thickness in 124 eyes with POAG (median MD: -3.9 dB).[12] Although the AUC of peripapillary vessel density measurement (0.83) was less than that of the average RNFL thickness (0.92), this difference was not statistically significant. Whole enface vessel density of the disc scan showed the best AUC in their study (AUC: 0.94), similar to that found in our study (0.93).[12] We found slightly greater AUCs of the peripapillary vessel densities and the RNFL thickness (compared to the results of the study by Yarmohammadi et al[12]) owing to eyes with more advanced glaucoma in our cohort (median MD: -6.3 dB). Additionally, we found that the AUC of average RNFL thickness was significantly greater than that of the peripapillary vessel density measurement. Glaucoma in our study was defined solely on the neuroretinal rim and RNFL changes on clinical examination and stereo photographs of the optic discs. This may have biased the diagnostic ability of the OCT rim area and RNFL thickness measurements and could have been the reason for the better diagnostic ability of structural measurements compared to vessel density measurements. We therefore ran a separate analysis considering optic disc changes and VF changes as the definition of glaucoma and found the results to be the same. Also, the diagnostic ability of macular measurements is less likely to be influenced by the reference standard. Therefore, the results of our study is likely to represent true superiority of structural measurements over vessel density measurements for diagnosing glaucoma. Future studies with functional tests as reference standard and longitudinal evaluation of suspect eyes are required to validate our results.

As expected, the diagnostic abilities of vessel densities and the structural measurements increased with increasing severity of glaucoma. This has been reported earlier both with vessel density[4, 13] and with structural measurements.[17] We therefore accounted for the severity of disease when evaluating for the effect of baseline IOP on the diagnostic abilities of vessel density and structural measurements. If reduced ONH blood supply was the predominant pathogenic mechanism in POAG eyes with low baseline IOPs (normal tension glaucoma, NTG), a greater difference in the vessel density values between the glaucoma and the control groups (and thereby a greater AUC) in these NTG eyes would be expected. However, this was not observed. On the contrary, the diagnostic ability of ONH vessel density increased in eyes with higher baseline IOP. This may imply that the vascular mechanisms contributing to the pathogenesis of glaucoma are not IOP-independent. IOP related stress and strain have been hypothesized to occlude the capillaries especially in the lamina cribrosa of the ONH.[24] Although there are no studies evaluating the effect of baseline IOP on the diagnostic abilities of the structural and vascular measurements of OCT as done in the current study, two previous studies have compared the diagnostic abilities of structural parameters of OCT in NTG (defined as open angle glaucoma eyes with baseline IOP<21 mm Hg) and POAG (defined as open angle glaucoma eyes with baseline IOP> = 21 mm Hg) patients with comparable glaucoma severity as defined on the VFs. These studies have found that the diagnostic ability of RNFL thickness and GCC thickness was greater in POAG compared to NTG.[25, 26] Contrary to the results of these studies, we found no statistically significant effect of the baseline IOP on the diagnostic abilities of RNFL and GCC thickness.

There are some limitations of the OCTA technology and the study design which need to be considered while interpreting the results. The vessel density measurements evaluated in this study were the ones provided by the software automatically. We therefore could not exactly match the vessel density sectors with the sectors of the structural parameters for comparison. The OCTA algorithm, in its current form, includes large vessels along with capillaries in its estimation of vessel density. The software also does not provide further insights into the nature of vascular changes such as attenuation, drop-out, etc. The technology also does not evaluate the choroidal vasculature. These details would provide a better understanding of the vascular changes in glaucoma. Another possible limitation of the current study was that we did not measure the blood pressure of the subjects or record their anti-hypertensive medication. However, we recorded the history of hypertension and found that the number of subjects with hypertension was similar in the glaucoma and the control groups. A previous study also has shown no relationship between blood pressure readings and peripapillary vessel densities on OCTA.[5] In the same context, the peripapillary vessel densities can also be affected by parapapillary atrophy (PPA).[27] We did not record the presence of PPA or its extent in our subjects. Another limitation of the study is the case-control design, with a clear distinction between glaucoma patients (cases) defined based on the presence of glaucomatous optic nerve head changes, and normal subjects (controls) with no suspicious findings of glaucoma. Such a design has been shown to overestimate the actual diagnostic ability of a test.[28–30].

In conclusion, we found that the diagnostic abilities of OCTA vessel density measurements of the ONH, peripapillary and the macular regions in POAG were significantly lower than the OCT ONH rim area, peripapillary RNFL thickness and the macular GCC thickness measurements, respectively. At fixed levels of glaucoma severity, the diagnostic ability of the OCTA ONH vessel density was significantly greater in eyes with higher baseline IOP. Baseline IOP did not affect the diagnostic ability of the other OCTA vessel density or the OCT structural measurements.
